# A Large-Scale Behavioral Screen to Identify Neurons Controlling Motor Programs in the *Drosophila* Brain

**DOI:** 10.1534/g3.113.006205

**Published:** 2013-10-01

**Authors:** Thomas F. Flood, Michael Gorczyca, Benjamin H. White, Kei Ito, Motojiro Yoshihara

**Affiliations:** * Department of Neurobiology, University of Massachusetts Medical School, Worcester, Massachusetts.; † Laboratory of Molecular Biology, National Institute of Mental Health, Bethesda, Maryland.; ‡ Institute of Molecular and Cellular Biosciences, University of Tokyo, Tokyo, Japan

**Keywords:** command neurons, *Drosophila*, Gal4, TRPM8, TrpA1

## Abstract

*Drosophila* is increasingly used for understanding the neural basis of behavior through genetically targeted manipulation of specific neurons. The primary approach in this regard has relied on the suppression of neuronal activity. Here, we report the results of a novel approach to find and characterize neural circuits by expressing neuronal activators to stimulate subsets of neurons to induce behavior. Classical electrophysiological studies demonstrated that stimulation of command neurons could activate neural circuits to trigger fixed action patterns. Our method was designed to find such command neurons for diverse behaviors by screening flies in which random subsets of brain cells were activated. We took advantage of the large collection of Gal4 lines from the NP project and crossed 835 Gal4 strains with relatively limited Gal4 expression in the brain to flies carrying a UAS transgene encoding TRPM8, a cold-sensitive ion channel. Low temperatures opened the TRPM8 channel in Gal4-expressing cells, leading to their excitation, and in many cases induced overt behavioral changes in adult flies. Paralysis was reproducibly observed in the progeny of crosses with 84 lines, whereas more specific behaviors were induced with 24 other lines. Stimulation performed using the heat-activated channel, TrpA1, resulted in clearer and more robust behaviors, including flight, feeding, and egg-laying. Through follow-up studies starting from this screen, we expect to find key components of the neural circuits underlying specific behaviors, thus providing a new avenue for their functional analysis.

The pioneering ethological study by Tinbergen and Lorenz (Tinbergen 1951) of the Graylag goose’s stereotypic behavior (a goose rolls a displaced egg back into the original incubating position) established the concept of a fixed action pattern for innate behaviors. A sequence of behaviors triggered by a certain sign stimulus, in this case an egg out of the nest (Tinbergen 1951), suggested that, at the neural level, a signal from visual sensory neurons is processed by interneurons that then trigger a stereotypic spatio-temporal pattern of motoneuron activity. A pivotal part of the neural circuits behind this process is the control switch that releases the pattern. The discovery of individual neurons in the crayfish central nervous system that could turn the rhythmic pattern of swimmeret movements on or off led to the concept of the command neuron (Ikeda and Wiersma 1964; Wiersma and Ikeda 1964), defined as an interneuron whose natural activity triggers a specific motor program.

Many such neurons have now been identified in diverse invertebrate animals, including mollusks, crustaceans, and insects, where they are involved in instinctive behaviors such as escape responses (Edwards *et al.* 1999; Frost and Katz 1996), feeding (Kupfermann and Weiss 2001; Marder and Calabrese 1996), and courtship songs (Bentley and Hoy 1974). Among the invertebrate systems for studying such behaviors, *Drosophila melanogaster* has special advantages because of the ease with which advanced genetic manipulation techniques can be applied. The ability to remotely control neuronal activity optogenetically by temporal regulation of ion channels by light (Lima and Miesenbock 2005) opened a new era in elucidating the function of brain circuits in freely moving and behaving animals. Lima and Miesenbock (Lima and Miesenbock 2005) activated neurons selectively in the giant fiber system, which is known to function in the *Drosophila* escape response (Allen *et al.* 2006), and induced escape behavior. However, the light activation techniques have technical limitations, such as the low penetration of light through the cuticle, which limit their effectiveness in certain situations. Recently, thermogenetic methods (Hamada *et al.* 2008; Peabody *et al.* 2009) have been developed to control neural activity with temperature-activatable ion channels, creating an effective way to activate neurons in large numbers of flies for genetic screening.

We took advantage of the Gal4 expression driver lines generated by the NP project (Yoshihara and Ito 2000) to screen for behaviors elicited by random neuronal stimulation. In *Drosophila*, the Gal4/UAS system (Brand and Perrimon 1993) allows one to drive transgenically introduced genes selectively in the Gal4-expressing cells. In each NP line, a Gal4 transgene has been inserted randomly into the fly genome, and Gal4 is reproducibly expressed in specific cells, depending on the activity of enhancer elements adjacent to the locus of insertion (O’Kane and Gehring 1987). Thus, by mating each Gal4 line to flies carrying a UAS transgene encoding a thermogenetic Trp channel, we can express these channels in various neurons in the brain and analyze the effects in freely behaving animals after channel activation by temperature shifts.

Two temperature-sensitive cation channels capable of activating targeted neurons have been developed for *Drosophila*: a cold-activatable channel from rat sensory neurons (TRPM8) (McKemy *et al.* 2002) and a heat-activatable channel from *Drosophila* thermosensory neurons (TrpA1) (Hamada *et al.* 2008). Activation of TRPM8 at reduced temperatures has been shown to generally stimulate fly neurons and has been shown to induce the behavioral program for wing expansion when selectively targeted to a single pair of neurons (Luan *et al.* 2012). Similarly, UAS constructs of TrpA1 can be used as a tool for thermogenetic activation of neurons for behavioral studies (Hamada *et al.* 2008). Here, we report the procedures and results of our screening of NP lines by observation of induced behaviors by neuronal activation using TRPM8 and TrpA1 in freely moving flies.

## Materials and Methods

### Fly strains

*Drosophila* crosses were performed at 21° or 25° according to standard protocols. Canton-S was used as the wild-type control. The NP (Nippon) Gal4 enhancer trap lines derive from insertion of Gal4 from the vector, pGawB (Brand and Perrimon 1993), into isogenized X, second and third chromosomes by the NP consortium (Yoshihara and Ito 2000). Chromosomes with a Gal4 insertion were balanced with *FM7c*, *CyO*, *TM3*, or *TM6* chromosomes in a background containing the isogenized chromosomes. *UAS-TRPM8* has been previously described (Peabody *et al.* 2009). *UAS-TrpA1* (Hamada *et al.* 2008) was a gift from P. Garrity. *UAS-GFP* S65T (T2 strain) was a gift from B. Dickson.

### TRPM8 screening procedure

Female transformants of UAS-TRPM8 (Peabody *et al.* 2009) were crossed to males of Gal4 lines established by the NP consortium (Yoshihara and Ito 2000) and reared at 25°. Three-day-old to 14-day-old progeny were tested in a custom-built plastic chamber with a double-layered ceiling, which enabled the temperature gradient to be maintained within ±1° from the floor to the ceiling (height, 4 mm) at experimental temperatures. For video recording, the arena was restricted to 19 mm × 15 mm to limit wandering. The chamber was designed to fit snuggly into a Nunc 35-mm plastic dish, and temperature was regulated by a TS-4 SPD Controller (Physitemp) and monitored with an IT-23 probe connected to a microprobe thermometer (BAT-10; Physitemp). Behavior was viewed and recorded using a dissection microscope (Stemi, 2000-c; Zeiss) with attached CCD camera. During testing, approximately 10 flies were introduced into a 15° chamber. Flies were observed for 1 min at 15°, then the temperature was lowered to 14° and the flies were observed for an additional 1 min while being recorded. For all flies showing induced behavior, the temperature was immediately increased to 25° after the 2 min at low temperature, and behavior was observed for an additional 2 min. This functioned as one type of negative control, because the TRPM8 channel has been shown to be inactive at 25° (Peabody *et al.* 2009).

### TrpA1 screening procedure

UAS-TrpA1 virgin females were crossed with males of various NP lines and reared at 21°. During this screening, 3-day-old to 6-day-old progeny were tested in the same chamber as that used for TRPM8 screening. During testing, approximately 10 flies were introduced into a 31° chamber and flies were observed for 2 min while being recorded. For all flies showing an induced behavior, after the 2-min observation was over, the temperature was immediately decreased to 21° and their behavior was observed for an additional 2 min. This functioned as a negative control, because the TrpA1 channel should be inactive at 21° (Hamada *et al.* 2008; Pulver *et al.* 2009).

### Video imaging

Videos were recorded from a CCD color camera (DFK31AF03; 1024 × 768 pixels; 1/3-in Sony CCD, ImagingSource). Videos were acquired at 15 or 30 frames per second.

### Statistics

Statistical analyses were performed according to established methods (Zar 1999).

## Results

### Genetic screen with TRPM8

We first examined gross expression patterns in the brain of approximately 2000 of the 3939 NP Gal4 enhancer trap lines (Yoshihara and Ito 2000) and chose 835 lines that had relatively limited Gal4 expression (*i.e.*, approximately 1000 cells or less), as revealed by anti-GFP staining of progeny from a cross to flies bearing a *UAS-GFP* transgene. The actual numbers of neurons with Gal4 expression were not quantified because this preselection screen was conducted primarily to exclude lines with broad expression patterns and thus to facilitate the later identification of neurons whose activation correlated with induced behaviors. For examples of lines selected for further behavioral screening together with examples of excluded lines, see Supporting Information, Figure S1. The 835 Gal4 lines selected for screening were crossed with flies carrying the UAS-TRPM8 transgene, and the progeny of crosses to 108 lines exhibited induced behaviors when tested at 15°, a temperature known to activate the TRPM8 (Mckemy *et al.* 2002; Peabody *et al.* 2009). After testing at 15°, the same flies were immediately retested at 25°, a temperature at which the TRPM8 channel is inactive (Peabody *et al.* 2009). No flies displayed the cold-induced (*i.e.*, 15°) behavior when retested at 25°. Further, the induced behavior at 15° appeared quickly, and increasing the temperature to 25° made the induced behavior disappear within approximately 30 sec, although most behaviors turned off with less delay. Additionally, wild-type flies did not show an induced behavior when tested at 15° (File S1). Together, these results strongly suggest that the behavioral induction is dependent on activation of the temperature-dependent TRPM8 channel.

**Figure 1:**
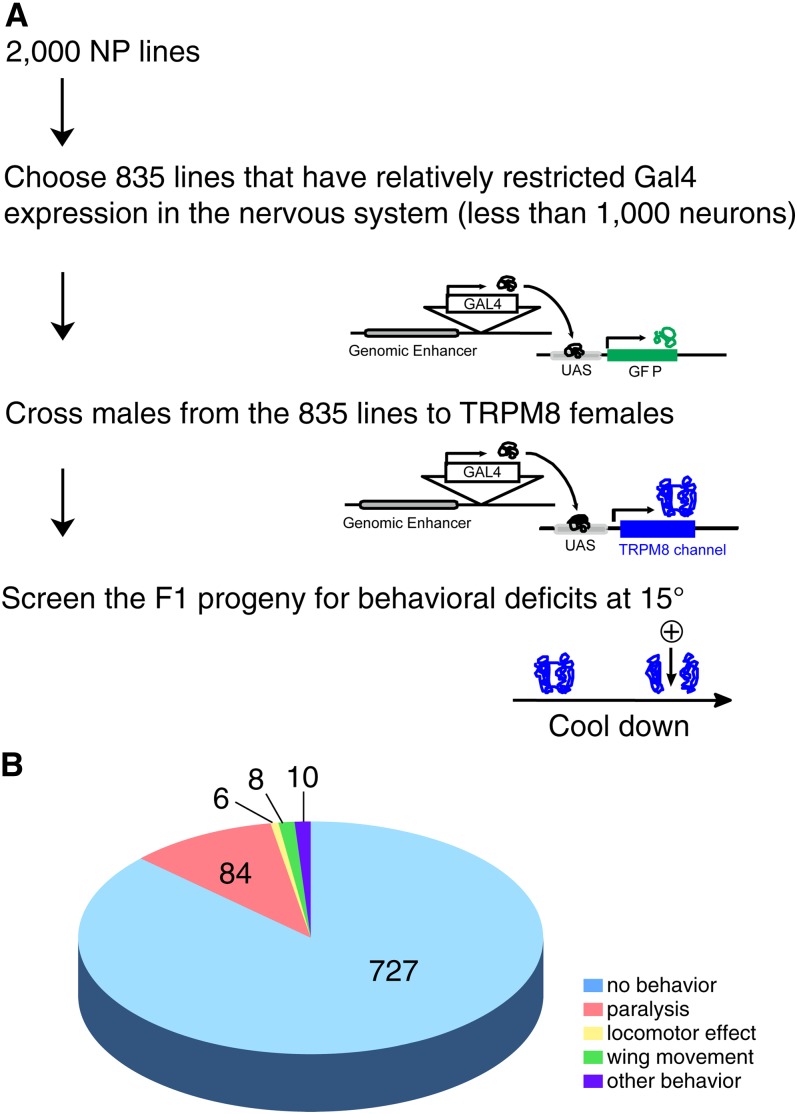
Protocol for TRPM8 screening and TRPM8-induced behaviors. (A) Protocol for behavioral screening using TRPM8. (B) Behaviors consisted of paralysis, changes in locomotion, wing movements, and various other actions. Pie chart summarizes categories of TRPM8-induced behaviors observed in screen. Number of NP lines identified for each category is indicated.

The TRPM8-induced behavioral changes were characterized and placed into several groups. These included paralysis, altered locomotion, wing movements, and various other patterns as described (Figure 1B). In all cases, the TRPM8-induced motor patterns were penetrant and repeatable. Some resembled natural behavioral sequences, whereas others appeared to be only fragments of such sequences.

### Paralysis

**Figure 2:**
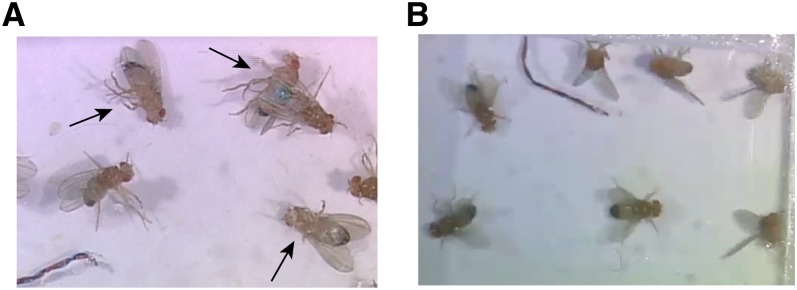
Representative TRPM8-induced behaviors. (A) Full paralysis is characterized by extreme postural instability and complete immobilization. Arrows mark immobile toppled flies, which are progeny of NP2106 × *UAS-TRPM8* cross at 15° (see also File S2). (B) The raised-wing phenotype is characterized by bilateral wing elevation and splaying. Flies are progeny of a NP377 × *UAS-TRPM8* cross at 15° (File S7).

TRPM8-induced paralysis was evident in three main forms, labeled in Table 1, as full paralysis (observed in 20 Gal4 lines), wing-beat paralysis (27 lines), and upright paralysis (37 lines). Full paralysis was characterized by extreme postural instability and complete immobilization (File S2; Figure 2A). Wing-beat paralysis was characterized by continual wing-beating with simultaneous postural instability and/or immobilization (File S3). Upright paralysis consisted of an upright immobilized fly without postural instability (File S4).

**Table 1:** TRPM8-induced paralysis in three dominant forms

Full Paralysis	Wing-Beat and Paralysis	Upright Paralysis
NP18	NP430	NP242
NP101	NP514	NP280
NP120	NP527	NP281
NP187	NP625	NP294
NP282	NP638	NP323
NP347	NP644	NP393
NP368	NP648	NP431
NP523	NP688	NP432
NP552	NP706	NP507
NP712	NP708	NP513
NP906	NP745	NP615
NP1128	NP777	NP681
NP1168	NP795	NP685
NP1183	NP808	NP696
NP1297	NP822	NP704
NP1542	NP830	NP740
NP1557	NP857	NP753
NP2106	NP887	NP812
NP2213	NP891	NP813
NP2355	NP894	NP829
	NP902	NP855
	NP974	NP864
	NP1137	NP903
	NP1164	NP912
	NP1198	NP933
	NP1201	NP1090
	NP2064	NP1106
		NP1221
		NP1284
		NP2045
		NP2147
		NP2311
		NP2358
		NP2360
		NP2366
		NP2376
		NP2411

From left to right, full paralysis (n = 20), wing-beat paralysis (n = 27), and upright paralysis (n = 37). See text for description of each. NP lines identified displaying each form of paralysis are listed in columns.

### Altered locomotion

Locomotor effects induced by TRPM8 activation consisted of two groups labeled “klutzy climbers” (two lines) and “tipsy” (four lines) (Table 2). Klutzy climbers were characterized by short intermittent seizures, which resulted in tumbling of the fly (File S5). Tipsy flies were characterized by slow, uncoordinated locomotion (File S6).

**Table 2:** Locomotor effects induced by TRPM8 activation consisted of two groups

Klutzy Climbers	Tipsy
NP35	NP115
NP2309	NP206
	NP212
	NP1208

From left to right, klutzy climbers (n = 2) and tipsy (n = 4). See text for description of each. NP lines identified displaying each form of locomotor effect are listed in columns.

### Wing movements

TRPM8-induced wing movements were categorized into three groups, wing-raise (two lines), wing-scissoring (two lines), and wing-beat (four lines) (Table 3). The wing-raise phenotype was characterized by bilateral wing elevation to approximately 45° to the horizontal axis of the body (File S7; Figure 2B). This behavior closely resembled wing-raising that occurs during initiation of voluntary flight (Card and Dickinson 2008) and also during aggressive displays (Kravitz and Huber 2003). Both sexes show the wing-raise behavior, although it was more obvious in males than in females. Wing-scissoring consisted of a quick scissoring of the wings, the planes of which remained parallel to the horizontal axis of the body (File S8). Wing-beat referred to continual beating of the wings without prominent postural instability, as seen in wing-beat paralysis (File S9).

**Table 3:** TRPM8-induced wing movements were categorized into three groups

Wing-Raise	Wing-Scissoring	Wing-Beat
NP210	NP502	NP271
NP377	NP635	NP437
		NP1241
		NP1609

From left to right, wing raise (n = 2), wing scissoring (n = 2), and wing beat (n = 4). See text for description of each. NP lines identified displaying each form of wing movement are listed in columns.

### Complex behavioral programs resembling natural behavior

Finally, some TRPM8-induced motor patterns resembled wild-type behavioral acts such as aggression (one line), grooming (two lines), exploration (two lines), and jumping (five lines) (Table 4). *Drosophila* aggression includes violent and intimidating acts against conspecifics such as wing threats, fencing, boxing, and chasing. Further, wing threats can be combined with a forward thrust to chase a rival away (Kravitz and Huber 2003). A pattern resembling this latter act was identified in the screen (File S10). It is important to note that although chasing is a sex-specific act occurring only in wild-type males, the video demonstrates induction of the behavior in both sexes.

**Table 4:** TRPM8-induced behaviors resembling wild-type behavior

Aggression-like	Grooming	Restless	Jumping
NP22	NP895	NP939	NP510
	NP1245	NP1144	NP957
			NP1603
			NP1629
			NP248

From left to right, aggression-like (n = 1), grooming (n = 2), exploration (n = 2), and jumping (n = 5). See text for description of each. NP lines identified displaying each form of induced behavior are listed in columns.

Grooming consists of cleaning components of the head, thorax, and abdominal segments with coordinated movements of the legs (Phillis *et al.* 1993). We identified TRPM8-induced repetitive grooming only by forelegs (File S11).

Wild-type fruit flies are known to explore their environment to locate valuable resources such as food, mates, and egg-laying sites (Tinette *et al.* 2007; Yang *et al.* 2008). We identified a behavior resembling fly exploration (File S12) in which TRPM8-induced flies continuously wandered without resting for the duration of the assay, which was atypical compared to a wild-type control (File S1) or other screened Gal4 strains.

Jumping occurs at initiation of voluntary flight and the execution of the escape response (Card and Dickinson 2008). We identified TRPM8-induced jumping (File S13). Wing elevation did not precede jumping, which would occur in initiation of flight, but rather the wings remained nestled against the fly’s body, reminiscent of the escape response (Card and Dickinson 2008).

### Genetic screen with TrpA1

Based on the screening results using TRPM8, we discarded the lines that showed relatively simple behavior (*e.g.*, paralysis) and retested the remaining lines using a similar protocol with the heat-sensitive cation channel TrpA1. TrpA1 has been reported to strongly induce action potentials (Hamada *et al.* 2008), and we reasoned that this channel might induce more robust responses than TRPM8 and ones that more clearly resembled innate behaviors. Thirty-two strains were tested, including several lines that had showed induced behavior on first screening but turned out to be false-positives in a reconfirmation test using TRPM8. The phenotypes observed in the tests with UAS-TrpA1 are detailed.

### Paralysis

Nine lines showed paralysis (Table 5), although they did not show such a phenotype with TRPM8, probably because the degree of neuronal activation was different between TRPM8 and TrpA1.

**Table 5:** TrpA1-induced paralysis

Full Paralysis	Wing-Beat and Paralysis
NP210	NP658
NP271	
NP514	
NP635	
NP785	
NP923	
NP939	
NP1241	

From left to right, full paralysis (n = 8) and wing-beat paralysis (n = 1). NP lines identified displaying each form of paralysis are listed in columns.

### Simple pattern of changes in locomotion or wing movements

**Figure 3:**
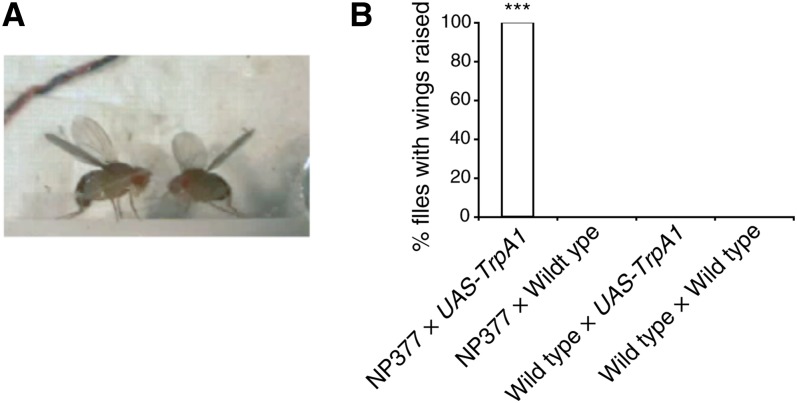
TrpA1-induced wing-raise. Wing-raise is characterized by bilateral wing elevation with medial rotation and may comprise part of the motor program for an aggressive display and/or initiation of flight. (A) Flies from the strain shown (*i.e.*, NP377) displayed the same TrpA1-induced wing phenotype as when tested with TRPM8 (see Figure 2B). Progeny were from a NP377 × *UAS-TrpA1* cross, shown at 31° (File S14). (B) Quantification of wing elevation at 31°. Contingency was tested by chi-square test and the significant difference between groups was found to be *P* < 0.0001. Because there was no significant difference between the three control groups, they were grouped as one and compared with NP377 × *UAS-TrpA1* females by Fisher exact test, resulting in a significant difference of *P* < 0.0001 (***; n = 20 for each genotype). To be scored as a positive wing-raise, a fly’s wings had to remain continuously elevated, as seen in (A), for more than 5 sec during a 5-min observation. Multiple flies were tested together (File S14).

As in the case of TRPM8, there were lines showing simple patterns such as wing raise (NP114, NP377, and NP502, which showed TRPM8-induced wing scissoring; File S14), airplane-like wing extension (NP22, which showed TRPM8-induced aggression; File S15), backstroke (NP1118; File S16), and crazy leg paralysis (NP523; File S17 and Table 6). Additional information about each behavior can be found in each Movie legend. Surprisingly, only one strain, NP377, displayed the same phenotype (wing raise) with TrpA1 as when tested with TRPM8 (Figure 2B and Figure 3, A and B; File S14). As discussed in greater detail in the Discussion, two things may explain the behavioral differences observed with the two channels. First, the expression levels and degrees of neuronal activation may differ between TRPM8 and TrpA1, potentially resulting in differential activation of neuronal networks. Second, the two temperatures used in the activation experiments (*i.e.*, 15° and 31°) may themselves differentially affect global neural excitability such that induction of activity in specific neurons results in different behavioral patterns (see Discussion).

**Table 6:** TrpA1-induced simple patterns of locomotion or wing movements

Wing-Raise	Airplane	Backstroke	Crazy Leg Paralysis
NP114	NP22	NP1118	NP523
NP377			
NP502			

From left to right, wing-raise (n = 3), airplane (n = 1), backstroke (n = 1), and crazy leg paralysis (n = 1). See text for description of each. NP lines identified displaying each form of induced behavior are listed in columns.

### Complex behavioral programs resembling natural behavior

Three categories of induced behaviors resembling components of wild-type instinctive behavior were identified using these lines, including egg laying, initiation of flight, and feeding as detailed (Table 7).

**Table 7:** TrpA1-induced behaviors resembling wild-type behavior

Egg-Laying	Feeding	Initiation of Flight
NP120	NP883	NP761
NP406		

From left to right, egg-laying (n = 2), feeding (n = 1), and initiation of flight (n = 1). See text for description of each. NP lines identified displaying each form of induced behavior are listed in columns.

### Egg-laying

**Figure 4:**
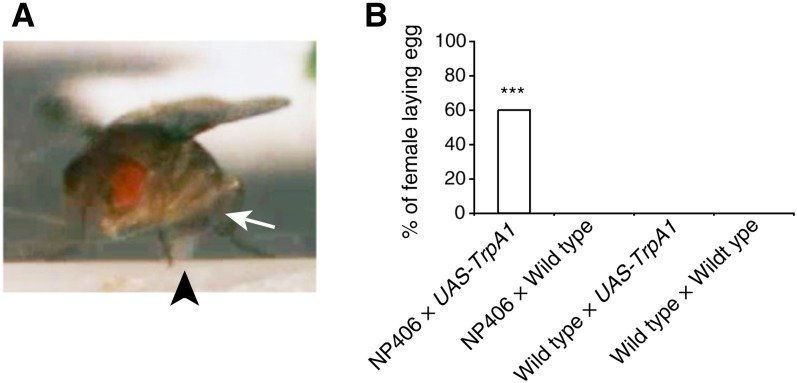
A TrpA1-induced behavior consisting of abdominal bending and egg expulsion, which resembles normal egg-laying behavior. (A) NP406 × *UAS-TrpA1* female fly displaying artificially induced abdominal bending (arrow) and egg expulsion (arrowhead) at 32°. (B) Quantification of egg-laying by female flies at 32°. Contingency was tested by chi-square test and the significant difference between groups was found to be *P* < 0.0001. Because there was no significant difference between the three control groups, they were grouped as one and compared with NP0406 × *UAS-TrpA1* females by Fisher exact test, resulting in a significant difference of *P* < 0.0001 (***; n = 20 for each genotype). To be scored positive, the egg must have protruded halfway or more out of the ovipositor during the 2-min observation period. Solitary flies were tested (File S18 and File S19).

Egg-laying behavior in *Drosophila* consists of a fixed sequence of relatively stereotyped motor patterns (Yang *et al.* 2008). A female fruit fly will search the environment and assess its quality by probing the surface with her proboscis, ovipositor, and legs. Once a suitable site is determined, the ovipositor motor program will commence. The ovipositor motor program consists of series of stereotyped motor patterns, such as bending of the abdomen, ovipositor substrate insertion, and egg ejection. After egg deposition, the fly will invariably groom its ovipositor. This final cleansing act completes the full sequence of egg-laying behavior. TrpA1-induced egg-laying was identified in the NP406 line (File S18 and File S19). The TrpA1-induced behavior consisted of abdominal bending and egg expulsion and resembled the wild-type behavior (Figure 4A). The induced egg-laying behavior occurred in the NP406-*Gal4*;*UAS-TrpA1* flies at 31°, but not at 21°, nor in control animals (Figure 4B). From these results, we conclude that TrpA1 activity induces abdominal bending and egg expulsion in females. Additional components of egg-laying behavior, such as the search and groom sequence described, were not obvious at 32°, at which temperature egg-laying was facilitated compared with 31°. Notably, the abdominal bending component of this behavior also was observed in males. We identified another line, NP120, which showed robust TrpA1-induced abdominal bending (File S20). In NP120, abdominal bending occurred only in female flies, as opposed to NP406. This apparent sexual dimorphism likely has a trivial explanation in that the Gal4 insertion site of NP120 is on the X chromosome and NP120 males were used for all crosses. In females, which exhibited the behavior, egg expulsion was not seen at 31°.

### Feeding

An animal’s feeding behavior consists of a collection of diverse behaviors such as foraging, recognition of food, and food ingestion. In response to an appropriate signal at the tarsal gustatory receptors on the legs, a starved wild-type fly will arrest locomotion, extend its proboscis, contact and taste a potential source of nourishment, and then retract the proboscis (Dethier 1976). The fly will reiterate this process until sated. Interestingly, NP883-*Gal4*/*UAS-TrpA1* flies displayed an induced behavior that represents the entire wild-type feeding sequence (File S21 and File S22). The origin of this phenotype is described elsewhere (Flood *et al.* 2013).

### Initiation of flight

**Figure 5:**
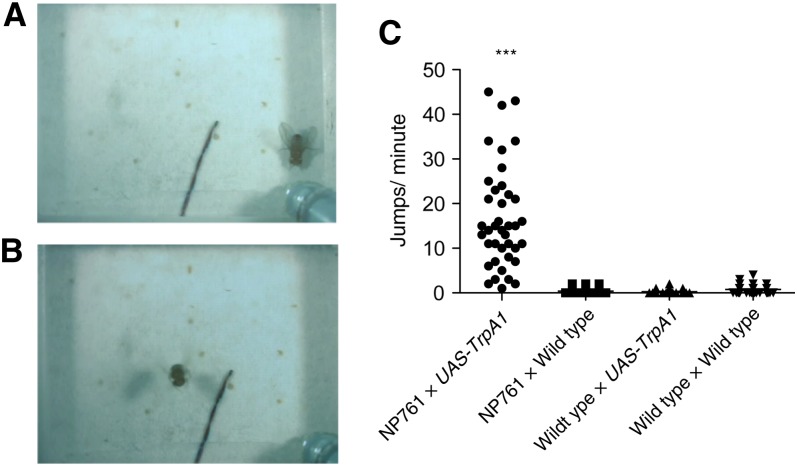
TrpA1-induced behavior resembling initiation of voluntary flight. During initiation of voluntary flight, a fly first raises its wings and then contracts its middle leg muscles, which propels the fly into the air, while simultaneously performing a down stroke. Once airborne, continuous wing-beating commences. We identified strain NP761, which demonstrated TrpA1-induced wing-raising, jumping, and wing-beating that together resemble initiation of voluntary flight. (A) Wing-spreading and elevation preceding jump. (B) A fly in actual flight within the 4-mm-high observation chamber. Progeny were of NP761 × *UAS-TrpA1* at 31°. (C) Progeny of NP761 × *UAS-TrpA1* displayed a significant increase in jumping relative to control animals (***P < 0.001 by Turkey multiple comparison test after a one-way ANOVA with *P* < 0.0001 significance). To be scored a positive jump, a fly must make a sudden horizontal translocation of >5 mm (approximate), excluding walking. Jumps were scored for a 1-min observation at 31°. Single flies were tested (n= 40 animals) (File S23 and File S24).

During initiation of voluntary flight, a fly first raises its wings and then contracts its middle leg muscles, which propels the fly into the air while simultaneously performing a down stroke (Card and Dickinson 2008). Once airborne, continuous wing-beating commences. This coordinated and relatively stereotyped sequence ensures a smooth and stable takeoff. We identified strain NP761, which demonstrated a TrpA1-induced behavior resembling initiation of voluntary flight (File S23). Interestingly, all components of the wild-type behavioral sequence appeared to be present in progeny of NP761-*Gal4* × *UAS-TrpA1* flies at 31°. This included wing elevation, jumping, and continuous wing-beating. Impressively, even actual flight was induced (File S24; Figure 5, A and B). No flight induction was observed when flies were tested at 21° (data not shown), nor was it observed in control animals (Figure 5C). Thus, we conclude that TrpA1 activity induced the expression of the motor patterns associated with initiation of flight.

With TRPM8, the lines mentioned in this section, except for NP883, showed only subtle behavioral alterations, reflecting a stronger effect of TrpA1. NP883 showed foreleg tapping with TRPM8, which also was observed in NP883 > TrpA1 flies along with feeding behavior (see Discussion).

## Discussion

### Behavioral screening by ectopic activation of genetically engineered channels

Our genetically targeted neuronal screen identified numerous artificially induced behaviors that closely resembled full or partial components of wild-type behavioral acts such as feeding, flight, courtship, and egg-laying. Importantly, the induced behaviors observed in 24 of the lines screened were quickly triggered and coordinated, suggesting that at least some were under command neuron control. These results suggest that the behavioral screening method we present here, which permitted the observation of freely moving flies, can sample a wide range of behaviors. This screen can then be used as a starting point to identify key neurons involved in specific behaviors by activating thermogenetic channels only in small subsets of neurons. We already have succeeded in identifying a putative command neuron for feeding behavior from the NP883 line (File S21 and File S22) using the flip-out Gal80 technique to create mosaic flies with significantly restricted numbers of expressing cells (Flood *et al.* 2013). The Fdg (feeding) neuron can induce the entire sequence of complex feeding behavior, and it was activated only when the fly was starved, indicating that it was involved in making the feeding decision depending on the fly’s state of satiety. In addition, some of our results indicate that screens, such as the one presented here, will be valuable in addressing a broader range of issues related to the organization of behavioral circuits.

### Comparison between TRPM8 and TrpA1

One of the most interesting observations made in our screen relates to the differing effects of activation by TRPM8 and TrpA1. As described in the Results, our initial screen using the cold-activated TRPM8 channel yielded 45 lines, 24 of which induced relatively specific behaviors. When UAS-TrpA1 flies became available, we rescreened these behavior-positive lines using this heat-activated channel. Somewhat surprisingly, we found that with one exception, NP377, the two channels induced different behaviors. This difference in behavioral output indicates that the two channels almost certainly produce different patterns of activity in the set of targeted neurons within each line’s expression pattern. This may be attributable, in part, to the efficacy in altering excitability of the two channels, which differ in numerous properties, including conductance, Ca^2+^ permeability, and desensitization, as well as temperature sensitivity. Compared with the relatively mild generation of action potentials by TRPM8 (Peabody *et al.* 2009), TrpA1 activation is reported to generate action potentials at more than 50 Hz at 29° (Hamada *et al.* 2008). The robust activation by TrpA1 has been successfully used for triggering behaviors mimicking natural courtship (Kohatsu *et al.* 2011; von Philipsborn *et al.* 2011). The difference in action potential frequency may be one major reason why we observed different behavioral patterns when using TRPM8 or TrpA1.

It is also worth noting, however, that the different temperature sensitivities of TRPM8 and TrpA1 mean that experiments using the two channels are conducted in entirely different temperature regimes. Although the *Drosophila* nervous system is designed for robust operation over a range of temperatures, the temperatures used to activate TRPM8 and TrpA1 (*i.e.*, 15° and 31°, respectively) are at opposite extremes of the regime in which *Drosophila* are viable and fertile. Because nervous system activity will generally be decreased at the former temperature and increased at the latter, transitions to each of these temperatures is expected to differentially affect the electrical properties not only of the targeted neurons but also of downstream neural components involved in the production of the behaviors observed. This fact also may contribute to the differences in the character and robustness of the observed motor output when the two channels were used. Finally, we cannot exclude the possibility that differences in genetic background led to differences in behavior pattern because the *TRPM8* and *TrpA1* lines were established in different laboratories and do not have the same isogenized background as the NP Gal4 lines (see Materials and Methods).

The other conclusion that can be drawn from the divergent effects of TRPM8 and TrpA1 activation is that the expression patterns of many Gal4 lines include components of multiple behavioral circuits. This is true even when the lines have been preselected, as was performed here, to reduce the number of affected cells, and may explain, in part, the large number of lines that produced paralytic phenotypes in our initial screen with TRPM8, presumably because of the activation of conflicting behavioral programs. Interestingly, we did not observe such behavioral conflicts in most of the 32 nonparalytic lines tested with TrpA1, and activation tended to induce a behavioral program that was different from that observed with TRPM8. This observation implies that these programs were produced by a single multifunctional circuit whose output was differentially biased by activating different neurons within that circuit, or that inhibitory interactions between the circuits (or elsewhere in the nervous system) suppressed one behavioral circuit in preference over the other. The last explanation is consistent with the well-established phenomenon of behavioral hierarchies (Davis 1979) in which dominant behaviors prevent the execution of hierarchically lower behaviors to avoid conflicts in motor output. The first explanation, however, is consistent with a growing body of studies that indicate that behavioral networks are often multifunctional. It is interesting to note in this regard that even command neurons, capable of releasing one behavior when stimulated, also have been found to be active within circuits that produce other behaviors (Kristan 2008). Given this, the combined use of TRPM8 and TrpA1 may provide a means not only of identifying command neurons but also of dissecting the functional roles of individual neurons in multiple or multifunctional circuits. In addition, it may help determine the types of network interactions that prevent behavioral conflicts.

### Use of Gal4 expression driver lines for analyzing behavioral circuits

Our behavioral screening of the NP lines (Yoshihara and Ito 2000) illustrates a general and efficient methodology for identifying Gal4 lines that contain within their expression patterns circuit elements capable of inducing robust behaviors when activated. The expression patterns then can be analyzed to identify the circuit elements involved in producing those patterns of behavior, including putative command neurons. This methodology will benefit from the increasing numbers of expression driver strains that are being generated (Jenett *et al.* 2012), which will increase the collection of testable neurons. Because each strain tends to label a heterogeneous population of neurons, among which only a few are expected to be responsible for the induced behavior, a comparison between the observed behavioral phenotypes and the gross expression patterns of these lines may be difficult. However, as we have shown elsewhere (Flood *et al.* 2013), the responsible neurons can be isolated further by applying methods for restricted targeting and combining them with appropriate techniques for monitoring and manipulating neuronal functions.

### Wing-raise behavior

The wing-raise behavior that we observed closely resembled wing-raise that occurs during initiation of voluntary flight and also during aggressive displays (Card and Dickinson 2008; Kravitz and Huber 2003). NP377 showed wing-raise by TRPM8 (File S7) and by TrpA1 (File S14), although it was not associated with social interactions. Another example of wing-raising by TRPM8 (NP22; File S10) was accompanied with weak social interaction that somewhat resembles natural aggression behavior (Kravitz and Huber 2003). It is important to note that although chasing is a sex-specific act occurring only in wild-type males, the video demonstrates induction of the behavior in both sexes. The appropriate neural circuitry for aggressive behavior may be present in both genders (Nilsen *et al.* 2004), with circuit function regulated in a sex-specific way as previously postulated for male-specific courtship song (Clyne and Miesenbock 2008).

An alternative possibility is that our screening may not have activated neural circuits related specifically to aggression. The neural circuit underlying the TRPM8-induced wing-raise phenotype could be common to both wild-type behavioral acts and may represent the coopting of a previously evolved behavior, such as a component of flight initiation, to serve as an aggressive display, analogous to how some molecules serve divergent cellular roles (Shen and Cowan, 2010).

### Egg-laying

NP406 showed abdominal bending and egg-laying in females and abdominal bending in males (File S18 and File S19), whereas NP120 showed only the abdominal bending observed in natural egg-laying behavior and not egg deposition (Yang *et al.* 2008). Understanding the nature of this difference between the two strains may prove useful in dissecting the precise neural circuitry regulating the complex and sex-specific behavior of egg-laying.

### Initiation of flight

TRPM8-induced jumping in NP957 (File S13) may result from activation of the cellular network directing the expression of the escape response. The coordinated flight behavior in NP761 (Figure 5; File S23 and File S24) suggests the possible activation of command neurons for voluntary flight initiation, although it is also possible that flight results from sensory activation because this NP line has expression in mechanosensory neurons (Kamikouchi *et al.* 2006).

### The next step: searching for command neurons

The identification of the Gal4 lines in this study prompted us to undertake further investigations to try to determine the neurons responsible for the observed behaviors. In most cases, the number of Gal4-positive neurons in the expression patterns of lines that produced a given behavior did not allow us to identify individual neurons that might participate in generating the behavior. To identify neurons responsible for each behavior, we found it necessary to conduct mosaic analyses to correlate expression in identified neurons with induced behavior. Using the flip-out Gal80 technique (Struhl and Basler 1993) to create mosaic flies with substantially restricted numbers of TrpA1-expressing cells (Flood *et al.* 2013), we recently succeeded in identifying a single pair of command neurons for feeding behavior in the expression pattern of the NP883 line (File S21 and File S22). The identified Fdg neuron can induce the entire sequence of complex feeding behavior, and it responded to a sucrose stimulus only when the fly was starved, indicating that it was involved in making the feeding decision depending on the fly’s state of satiety. NP883 was initially isolated by the observation of foreleg tapping using TRPM8 activation, and the same tapping behavior also was observed with TrpA1 in addition to the feeding activity, a behavior not observed by TRPM8. The tapping behavior was segregated out from feeding behavior by the flip-out Gal80 technique, suggesting that the former is induced by the activation of other cell(s) expressed in the same line. The fact that we were ultimately able to isolate a single command neuron for feeding behavior validates the utility of the screening paradigm presented here for addressing a broad range of issues related to the functional organization of behavioral circuits.

### Strong potential of this method

Finally, it is worth noting that because the method presented here relies on the Gal4/UAS system, neurons identified to function in a behavioral circuit can be further characterized using available UAS transgenes in conjunction with the Gal4 driver used to target the neurons. The role of molecules in an observed behavior easily can be tested by expressing UAS rescue constructs in a mutant genetic background and/or by specific expression of RNAi constructs in putative command neurons. GFP and other fluorescent reporters can be used to visualize putative command neurons, allowing them to be identified for electrophysiological analysis and probed with electrodes. In addition, reporters targeted to various subcellular structures can be expressed in putative command neurons, allowing one to localize presynaptic and postsynaptic compartments (Flood *et al.* 2013) and to assess the direction of information flow through the neuron. Genetically engineered calcium indicators such as GCaMP (Nakai *et al.* 2001), or indicators of cAMP such as UAS-Epac-CaMPs (Shafer *et al.* 2008), can be introduced into the cell to image its function (Flood *et al.* 2013). Inputs or targets of a putative command neuron can be identified using genetic activation or inactivation of neurons suspected to synapse with the command neuron, leading to enhancement or suppression of command neuron function. The GRASP technique (Feinberg *et al.* 2008; Gordon and Scott 2009), which can reveal physical associations between two cells, also could be used in conjunction with the methods introduced here to elucidate neuronal circuitry. In this technique, a distinct fragment of GFP is expressed on the surface of each cell and fluorescence is produced only when both subunits are in close proximity. Other expression activation systems such as LexA enhancer-trap strains (Lai and Lee 2006; Miyazaki and Ito 2010) can be used to visualize neurons that are potentially in contact with Gal4-expressing neurons of interest. Labeling of synaptic structures (Flood *et al.* 2013), such as axon terminals or dendritic endings, during activation of an identified neuron would allow for time-lapse imaging of activity-dependent changes in synaptic structure and could be used to test potential mechanisms of synaptic plasticity suggested from studies of neuromuscular junctions (Yoshihara *et al.* 2005). Simultaneous imaging of synaptic structure and behavioral observation, as has been accomplished with feeding command neurons and proboscis extension behavior (Yoshihara 2012), could produce correlations between synaptic plasticity and memory as behavioral changes, because alteration in the strength of commanded behavior could be assayed as a manifestation of memory formation. Taken together, these methods can be applied in diverse ways to the *Drosophila* model system to gain insight into brain circuits and lead to a better understanding of information processing.

## Supplementary Material

Supporting Information
