# Supplementary material for: A Large-Scale Behavioral Screen to Identify Neurons Controlling Motor Programs in the Drosophila Brain
Source: G3 (Bethesda). 2013 Oct 1;3(10):1629–37. doi: 10.1534/g3.113.006205 (PMC3789788; doi:10.1534/g3.113.006205)
Supplement: Supporting Information [file supp_3_10_1629__index.html]

A Large-Scale Behavioral Screen to Identify Neurons Controlling Motor Programs in the Drosophila Brain — Supporting Information 

# A Large-Scale Behavioral Screen to Identify Neurons Controlling Motor Programs in the *Drosophila* Brain

## Supporting Information for Flood *et al.*, 2013

**Files in this Data Supplement:**

- Supporting Information - Figure S1 and Files S1-S24 (PDF, 722 KB)
- Figure S1 - Gal 4 expression patterns monitored with UAS-GFP in representative NP lines preselected for further behavioral screening (A) and NP lines excluded from the behavioral screening due to too many cells expressing Gal4 (B). (PDF, 569 KB)
- File S1 - Wild-type behavior at 15° (.avi, 4 MB)
- File S2 - TRPM8-induced 'Full Paralysis' (.avi, 4 MB)
- File S3 - TRPM8-induced 'Wing Beat Paralysis' (.avi, 2 MB)
- File S4 - TRPM8-induced 'Upright Paralysis' (.avi, 2 MB)
- File S5 - TRPM8-induced 'Klutzy Climbers' (.avi, 4 MB)
- File S6 - TRPM8-induced 'Tipsy' (.avi, 4 MB)
- File S7 - TRPM8-induced 'Wing Raise' (.avi, 3 MB)
- File S8 - TRPM8-induced 'Wing Scissoring' (.avi, 3 MB)
- File S9 - TRPM8-induced 'Wing Beat' (.avi, 3 MB)
- File S10 - TRPM8-induced 'Aggression' or 'Gregarious' (.avi, 4 MB)
- File S11 - TRPM8-induced 'Grooming' (.avi, 3 MB)
- File S12 - TRPM8-induced 'Restless' (.avi, 4 MB)
- File S13 - TRPM8-induced 'Jumping' (.avi, 4 MB)
- File S14 - TrpA1-induced 'Wing Raise' (.avi, 2 MB)
- File S15 - TrpA1-induced 'Airplane' (.avi, 2 MB)
- File S16 - TrpA1-induced 'Backstroke' (.avi, 2 MB)
- File S17 - TrpA1-induced 'Crazy Leg Paralysis' (.avi, 2 MB)
- File S18 - TrpA1-induced 'Egg Laying' (.avi, 2 MB)
- File S19 - TrpA1-induced 'Egg Laying' part 2 (.avi, 986 KB)
- File S20 - TrpA1-induced 'Abdominal Bending' (.avi, 2 MB)
- File S21 - TrpA1-induced 'Feeding' (.avi, 2 MB)
- File S22 - TrpA1-induced 'Feeding' part 2 (.avi, 2 MB)
- File S23 - TrpA1-induced 'Initiation of Voluntary Flight' (.avi, 2 MB)
- File S24 - TrpA1-induced 'Initiation of Voluntary Flight' part 2 (.avi, 709 KB)
