# Supplementary material for: A Large-Scale Behavioral Screen to Identify Neurons Controlling Motor Programs in the Drosophila Brain
Source: G3 (Bethesda). 2013 Oct 1;3(10):1629–37. doi: 10.1534/g3.113.006205 (PMC3789788; doi:10.1534/g3.113.006205)
Supplement: Supporting Information [file supp_g3.113.006205_006205SI.pdf]

G3·Genes | Genomes | Genetics

A large-scale behavioral screen to identify neurons controlling motor programs in the *Drosophila* brain

Thomas F. Flood*, Michael Gorczyca*, Benjamin H. White§, Kei Ito**, and Motojiro Yoshihara*

*Dept. of Neurobiology, Univ. of Massachusetts Medical School, Worcester, MA. § Laboratory of Molecular Biology, NIMH, Bethesda, MD. ** Institute of Molecular and Cellular Biosciences, University of Tokyo, Tokyo, Japan.

DOI: 10.1534/g3.113.006205

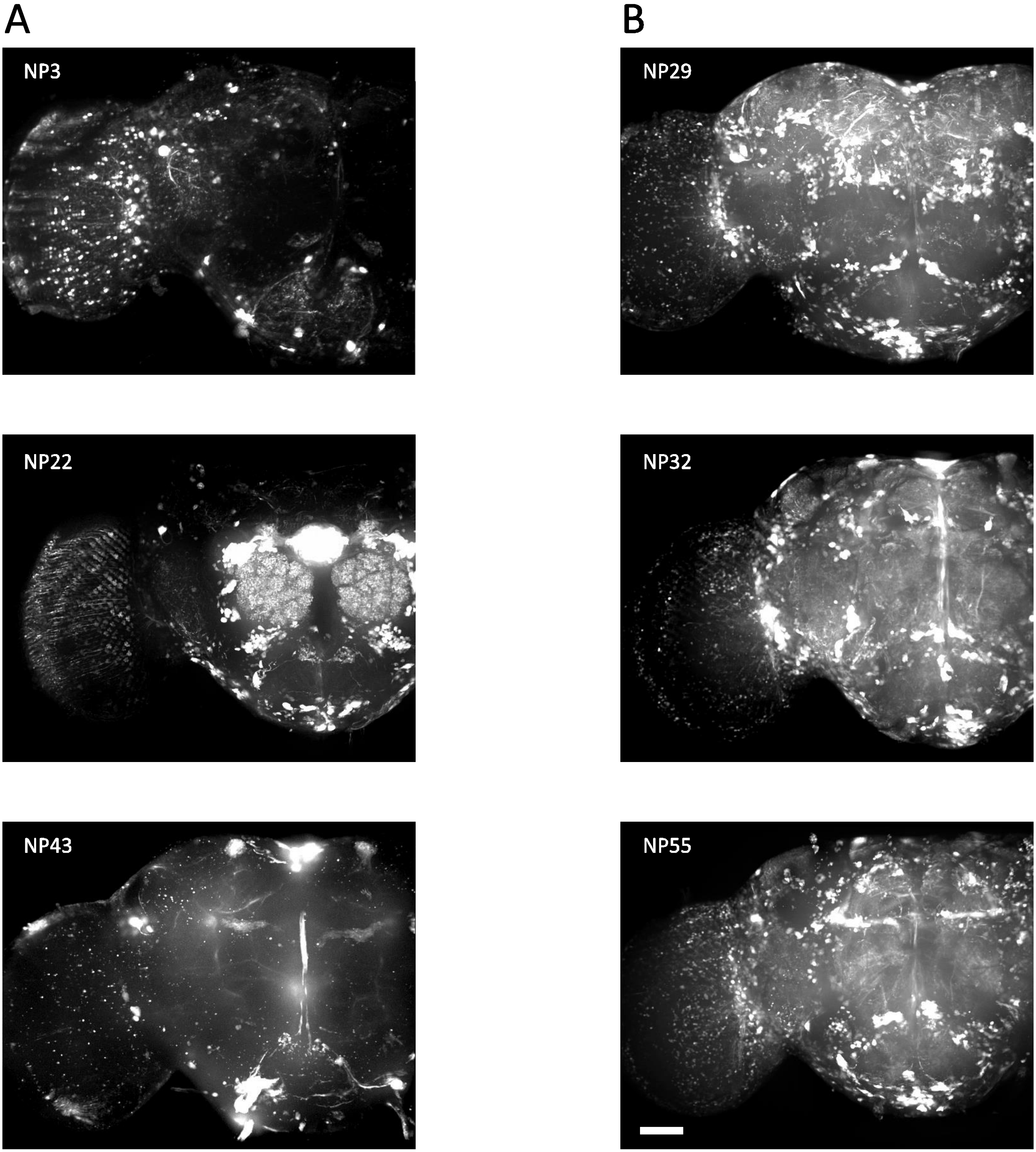

Figure S1 Gal 4 expression patterns monitored with UAS-GFP in representative NP lines preselected for further behavioral screening (A) and NP lines excluded from the behavioral screening due to too many cells expressing Gal4 (B). Scale bar, 50 µm.

Files S1-S24

Available for download as .avi files at http://www.g3journal.org/lookup/suppl/doi:10.1534/g3.113.006205/-/DC1.

File S1 Wild-type behavior at 15°. Canton S flies at 15° demonstrate normal behavior including environmental exploration, conspecific inspection and grooming. This video begins 30 seconds after the flies were exposed to 15°.

File S2 TRPM8-induced 'Full Paralysis'. Full paralysis is characterized by extreme postural instability and complete immobilization, as demonstrated in this video by flies toppling over and not moving. In video: progeny of NP2106 x *UAS-TRPM8* flies at 15°.

File S3 TRPM8-induced 'Wing Beat Paralysis'. Wing beat paralysis is characterized by continual wing beating, with simultaneous postural instability and/or immobilization. In video: progeny of NP648 x *UAS-TRPM8* flies at 15°.

File S4 TRPM8-induced 'Upright Paralysis'. 'Upright Paralysis' consists of an upright, mostly immobilized fly with postural stability. In video: progeny of NP2376 x *UAS-TRPM8* flies at 15°.

File S5 TRPM8-induced 'Klutzy Climbers'. 'Klutzy Climbers' is a phenotype characterized by climbing followed by falling onto the back and difficulty righting. In video: progeny of NP2309 x *UAS-TRPM8* flies at 15°.

File S6 TRPM8-induced 'Tipsy'. "Tipsy" is characterized by slow uncoordinated locomotion. A 'Klutzy Climbers' phenotype is also observed sometimes in flies scored with this phenotype. In video: progeny of NP1208 x *UAS-TRPM8* flies at 15°.

File S7 TRPM8-induced 'Wing Raise'. The "Wing Raise" phenotype is characterized by bilateral wing elevation. As seen in the video the wings are spread out and raised at forty-five degrees to the horizontal axis. Further, this video demonstrates the delayed induction of the behavior. In general all positive strains produce an induced behavior within 30 seconds of being at 15°. In video: progeny of NP377 x *UAS-TRPM8* flies at 15°.

File S8 TRPM8-induced 'Wing Scissoring'. Wing Scissoring consists of a quick scissoring of the wings, which remain parallel to the horizontal axis. Interestingly, this behavior is induced much more quickly than the 'Wing Raise' phenotype (File 7). In video: progeny of NP502 x *UAS-TRPM8* flies at 15°.

File S9 TRPM8-induced 'Wing Beat'. Wing Beat refers to continual bilateral beating of the wings without prominent postural instability. In video: progeny of NP1241 x *UAS-TRPM8* flies at 15°.

File S10 TRPM8-induced 'Aggression' or 'Gregarious.' In this phenotype, a wing raise can be combined with chasing another fly. In this video flies raise their wings and run throughout the chamber and appear to interact. Although this behavior is similar to "Wing Raise" of NP377, in this case the wings were not always raised, but seemed to be raised when interacting with another fly. In video: progeny of NP22 x *UAS-TRPM8* flies at 15°.

File S11 TRPM8-induced 'Grooming'. Grooming consists of cleaning parts of the body with coordinated movements of the legs. We identified TRPM8-induced repetitive grooming only by forelegs to clean the proboscis, the forelegs and the head. In video: progeny of NP1245 x *UAS-TRPM8* flies at 15°.

File S12 TRPM8-induced 'Restless'. This video shows the "Restless" phenotype in TRPM8-induced flies that continuously wandered for the duration of the assay, without stopping, resembling exploration. In video: progeny of NP1144 x *UAS-TRPM8* at 15°.

File S13 TRPM8-induced 'Jumping'. Successful jumping is necessary for initiation of voluntary flight and the execution of the escape response. We identified TRPM8-induced jumping. Wing elevation does not precede jumping, which would occur in initiation of flight, but rather the wings remained nestled against the fly's body reminiscent of the escape response. In video: progeny of NP957 x *UAS-TRPM8* at 15°.

File S14 TrpA1-induced 'Wing Raise'. One strain, NP377, displayed the same TrpA1-induced phenotype as when tested with TRPM8. The increased movement is most likely due to the elevated temperature. In video: progeny of NP377 x *UAS-TrpA1* at 31°.

File S15 TrpA1-induced 'Airplane'. 'Airplane' is a phenotype characterized by bilateral wing extension perpendicular to the length of the body. Further the wings are rotated 90 degrees. This behavior is very different from the TRPM8-induced behavior in the same strains (File 10), although both effectors induced wing movements. This line may be valuable for understanding the

neural control of the wings. In video: progeny of NP22 x *UAS-TrpA1* at 31°.

File S16 TrpA1-induced 'Backstroke'. Flies displaying the 'Backstroke' phenotype move around the chamber on their back, with wings slightly spread and legs flailing. In video: progeny of NP1118 x *UAS-TrpA1* at 31°.

File S17 TrpA1-induced 'Crazy Leg Paralysis'. The 'Crazy Leg Paralysis' phenotype is characterized by paralysis with continuously and vigorously flailing legs. Wings are spread out and flies are on their back. In video: progeny of NP523 x *UAS-TrpA1* at 31°.

File S18 TrpA1-induced 'Egg Laying'. Once a suitable egg laying site is determined the ovipositor motor program (OMP) will commence. The OMP consists of a series of stereotyped motor programs, including bending of the abdomen, ovipositor substrate insertion and egg ejection. The TrpA1-induced behavior consisted of abdominal bending and egg expulsion and resembled the wild-type behavior. The fly in the bottom right corner of the video demonstrates this well. In video: progeny of NP406 x *UAS-TrpA1* at 32°.

File S19 TrpA1-induced 'Egg Laying' part 2. Higher magnification video image of TrpA1a-induced abdominal bending and egg expulsion. Fly is upside down. In video: progeny of NP406 x *UAS-TrpA1* at 32°.

File S20 TrpA1-induced 'Abdominal Bending'. Robust TrpA1-induced abdominal bending. The Gal4 insertion in the NP120 line is X-linked. In video: progeny of NP120 x *UAS-TrpA1* at 31°.

File S21 TrpA1-induced 'Feeding'. In response to an appropriate gustatory stimulus a starved wild-type fly will arrest locomotion, extend its proboscis, contact and taste a potential source of nourishment, and then retract the proboscis. The fly will reiterate this process until sated. NP883 x *UAS-TrpA1* flies displayed repetitive proboscis extension/retraction resembling the wild-type feeding response. In video: progeny of NP883 x *UAS-TrpA1* at 31°.

File S22 TrpA1-induced 'Feeding' part 2. High magnification video image of a fly displaying repetitive proboscis extension/retraction. In video: progeny of NP883 x *UAS-TrpA1* at 31°.

File S23 TrpA1-induced 'Initiation of Voluntary Flight'. During initiation of voluntary flight a fly first raises its wings and then contracts its middle leg muscles to propel itself into the air, while simultaneously performing a down stroke. Once airborne, continuous wing beating commences. This coordinated and relatively stereotyped sequence ensures a smooth and stable take off. We identified strain NP761, which demonstrated TrpA1-induced wing raising, jumping, and wing beating that together resemble initiation of voluntary flight. In video: progeny of NP761 x *UAS-TrpA1* at 31°.

File S24 TrpA1-induced 'Initiation of Voluntary Flight' part 2. All components of the wild-type behavioral sequence appear to be present in the TrpA1-induced behavior, such as wing elevation, jumping, and continuous wing beating. Impressively, in this video actual flight is induced. In video: progeny of NP761 x *UAS-TrpA1* at 31°.
