# Supplementary material for: A Large-Scale Behavioral Screen to Identify Neurons Controlling Motor Programs in the Drosophila Brain
Source: G3 (Bethesda). 2013 Oct 1;3(10):1629–37. doi: 10.1534/g3.113.006205 (PMC3789788; doi:10.1534/g3.113.006205)
Supplement: Supporting Information [file supp_g3.113.006205_FigureS1.pdf]

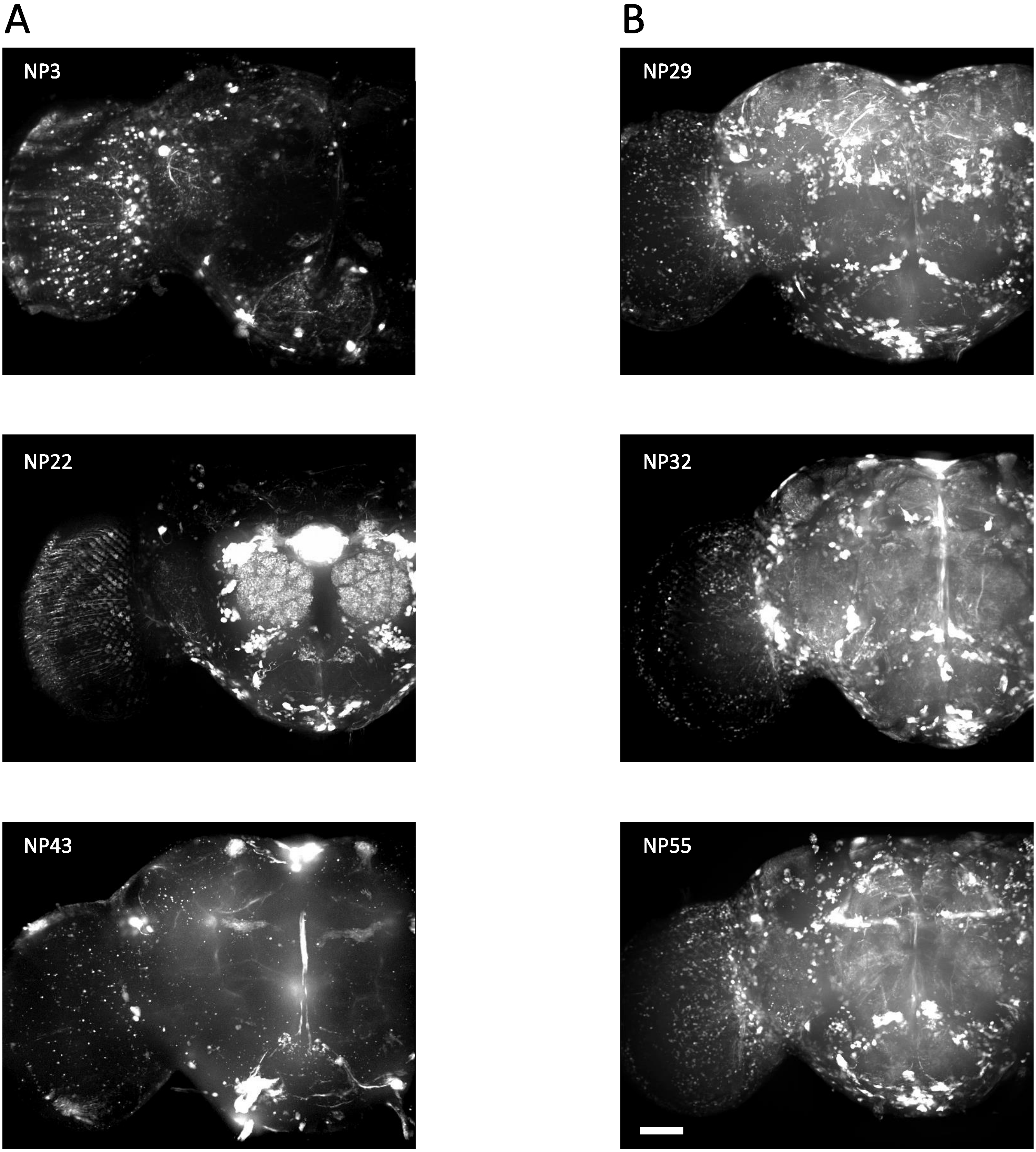

Figure S1 Gal 4 expression patterns monitored with UAS-GFP in representative NP lines preselected for further behavioral screening (A) and NP lines excluded from the behavioral screening due to too many cells expressing Gal4 (B). Scale bar, 50 µm.
